# Current research on ecotoxicity of metal-based nanoparticles: from exposure pathways, ecotoxicological effects to toxicity mechanisms

**DOI:** 10.3389/fpubh.2024.1390099

**Published:** 2024-07-15

**Authors:** Fang Wang, Li Zhou, Dehong Mu, Hui Zhang, Gang Zhang, Xiangming Huang, Peizheng Xiong

**Affiliations:** ^1^Department of Ophthalmology, Chengdu First People's Hospital, Chengdu, China; ^2^Department of Torhinolaryngology, Hospital of Chengdu University of Traditional Chinese Medicine, Chengdu, China; ^3^Department of Oncology, Chengdu Second People's Hospital, Chengdu, China; ^4^Department of Otorhinolaryngology, The First Affiliated Hospital of Guangxi University of Traditional Chinese Medicine, Nanning, China

**Keywords:** metal-based NPs, exposure pathway, toxic effects, toxicity mechanisms, review

## Abstract

Metal-based nanoparticles have garnered significant usage across industries, spanning catalysis, optoelectronics, and drug delivery, owing to their diverse applications. However, their potential ecological toxicity remains a crucial area of research interest. This paper offers a comprehensive review of recent advancements in studying the ecotoxicity of these nanoparticles, encompassing exposure pathways, toxic effects, and toxicity mechanisms. Furthermore, it delves into the challenges and future prospects in this research domain. While some progress has been made in addressing this issue, there is still a need for more comprehensive assessments to fully understand the implications of metal-based nanoparticles on the environment and human well-being.

## 1 Introduction

Metal-based nanoparticles (NPs) are metal-based particles with nanometric dimensions. Due to their exceptionally large specific surface area, these particles possess exceptional physicochemical properties, including catalysis, light absorption and magnetic properties (1–3). Metal-based NPs have diverse applications in electronic devices, energy storage, and conversion (4–6). For example, FeN_4_ graphite nanosheets show promise for improving oxygen electrocatalytic activity and durability in zinc-air batteries (7); and gold NPs (AuNPs), for the photothermal enhancement of tumor vascular destruction (8). Copper sulfide NPs are an inexpensive and widely available plasma material that exhibits high photothermal conversion efficiency, making it suitable for solar evaporation and water purification applications (9). Fe_7_Se_8_ NPs supported on nitrogen-doped carbon nanofibers are utilized as a high-rate anode material for sodium ion batteries (10).

However, there are also potential risks to the environment and human well-being associated with the widespread use of metal-based NPs. Metal-based NPs can be released into the environment during manufacture, use and disposal and then cause ecotoxicity through various exposure pathways (11). The ecotoxicity of metal-based NPs refers to their adverse effects on the survival, growth, and reproduction of organisms in the environment, including microorganisms, plants, and animals. The mechanisms of ecotoxicity include physical and chemical effects such as oxidative stress, DNA damage, and Cell membrane damage (12, 13). Research on the ecotoxicity of metal-based NPs is still in its infancy, and there are many challenges in the research process. The first challenge is how to measure the exposure of metal-based NPs to organisms. Metal-based NPs are difficult to measure due to their small size and aggregation properties. The second challenge is how to accurately assess the toxicity of metal-based NPs. Metal-based NPs have different toxicities in different organisms and under different conditions. Therefore, it is necessary to conduct toxicological experiments under controlled conditions to obtain accurate toxicity data.

**Figure 1:**
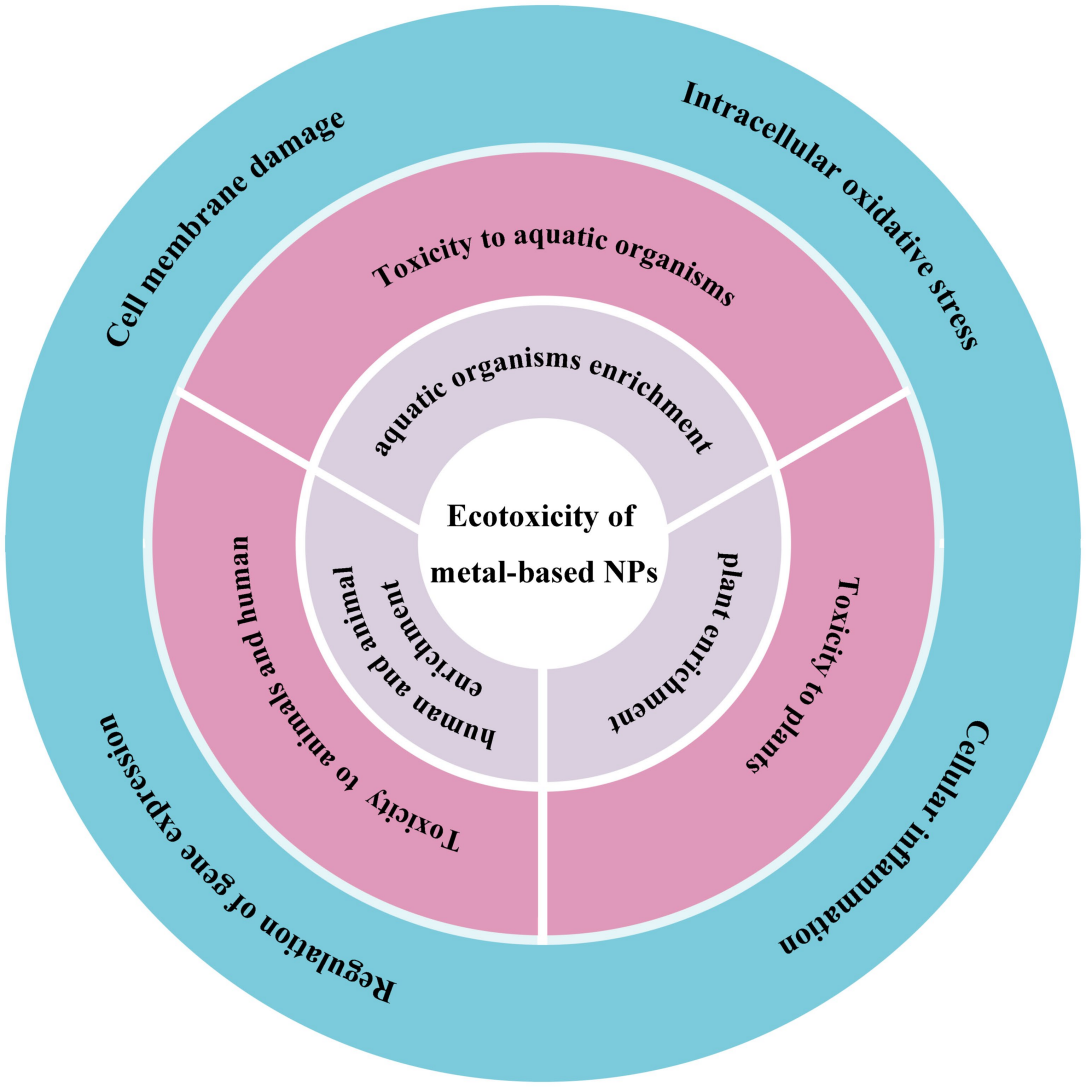
The schematic shows the ecotoxicity induced by metal-based NPs from the exposure pathways (grey), ecotoxicological effects (pink) to toxicity mechanisms (blue).

In this review, we summarize recent advances in ecotoxicity studies of metal-based NPs, including their exposure pathways, ecotoxicological effects and toxicity mechanisms. For metal-based NPs of natural origin, their toxicity may differ from that of synthetic NPs. Naturally occurring NPs are often encapsulated or stabilized by other substances found in nature, which may affect their biological activity and toxicity. In addition, natural NPs are often less concentrated and have evolved and dispersed in the environment over a long period of time, which may have reduced their potential toxicity. Since there are relatively few toxicity studies on natural metal-based NPs, we focus on the ecotoxicity of engineered metal-based NPs. We also discuss the challenges and prospects for ecotoxicity studies of metal-based NPs and how to comprehensively assess the impact of metal-based NPs on the environment and human health (Figure 1).

## 2 Exposure pathways to metal-based NPs

Due to the distinctive characteristics of NPs, their impact on organisms is expected to manifest through various exposure pathways (14). NPs are small in size and can thus pass through the cell membrane, cytoplasm, and nucleus, entering directly into the cell interior, making its mode of exposure significantly different from that of other particles (15–17). Generally, NPs enter the organism through absorption, diffusion, contact, and binding. This exposure mode can largely reflect the direct effects of NPs on organisms.

### 2.1 Exposure pathways of aquatic organisms enrichment

The enrichment exposure pathway of metal-based NPs in aquatic ecosystems is a matter of great concern. These NPs may have far-reaching effects on aquatic organisms and the entire ecosystem due to their unique physical and chemical properties.

**Figure 2:**
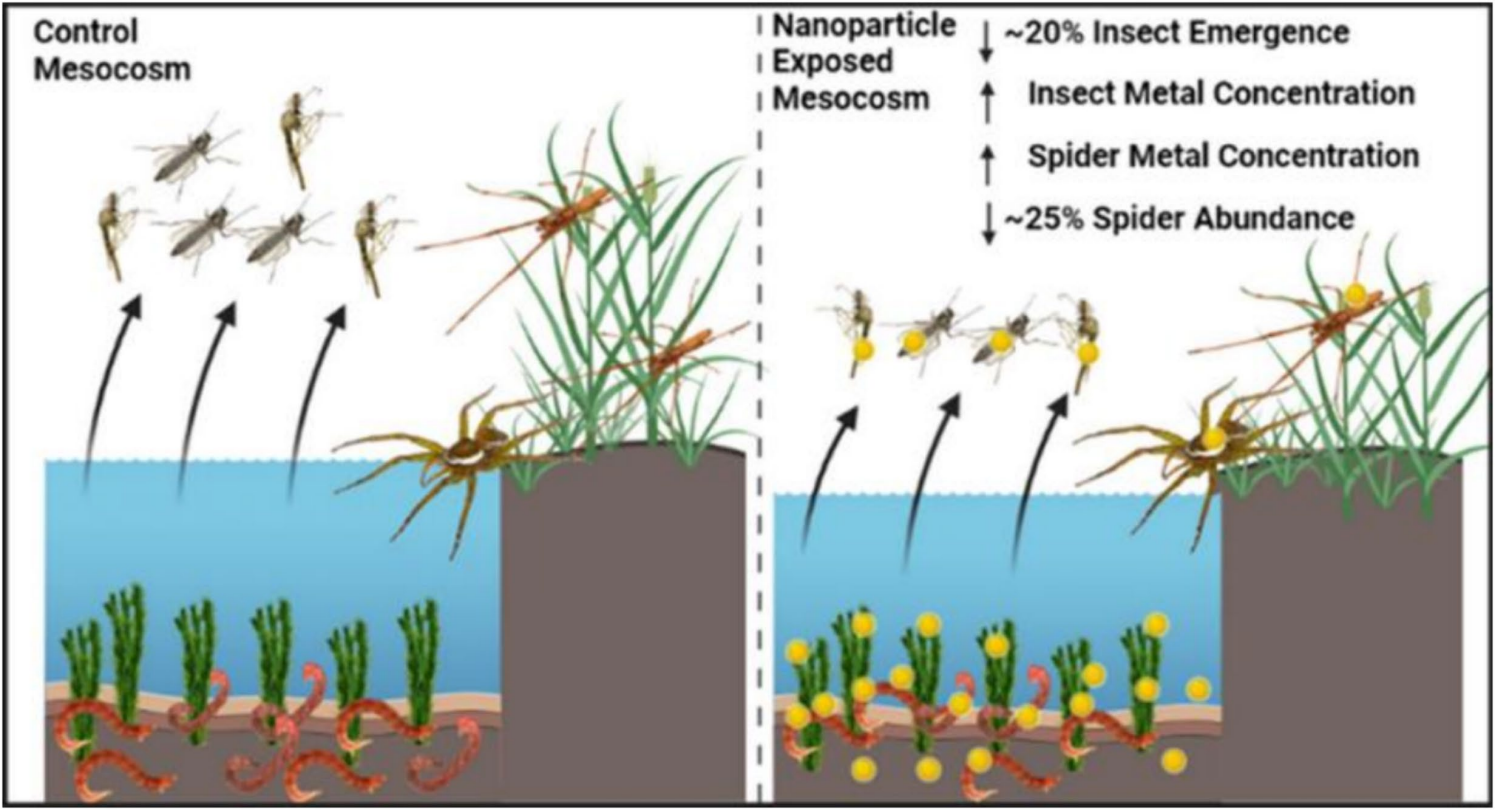
Schematic representation of the transfer of metal-based NPs from aquatic to terrestrial ecosystems (18). Copyright 2023, American chemical society.

First, metal-based NPs can enter aquatic organisms through direct contact. Metal-based NPs enter freshwater ecosystems through wastewater discharges and agricultural runoff. These NPs, such as copper and gold, can be taken up by tissues within aquatic organisms and accumulate, leading to the transfer of metals from aquatic to terrestrial ecosystems (Figure 2) (18). In addition, the presence of organic matter can influence the behavior and toxicity of metal-based NPs, for example, it can reduce the toxicity of AgNPs to bacteria and protozoa (19). This suggests that the bioaccumulation process of metal-based NPs is influenced by organic matter in the environment.

Metal-based NPs can also spread in aquatic ecosystems through biotransfer mechanisms. Biotransfer is the process by which one organism transfers substances from the environment to another organism (20). For example, AgNPs can be transferred and biomagnified to Tetrahymena thermophila through the food chain (19). In addition, the transformation, bioavailability, and toxic effects of metal-oxide-based NPs in fresh water on invertebrates suggest a potential risk of their delivery in the food chain (21).

Finally, the ability of metal-based NPs to bioaccumulate and biomagnify depends on a variety of factors, including the physicochemical properties of the NPs, the physiological properties of the organism, and environmental conditions. For example, studies of the accumulation dynamics of silver NPs with different coatings in simple freshwater food chains have shown that diet is the main uptake pathway for silver NPs (22). The ability of marine invertebrates to bioaccumulate heavy metals is also influenced by their physiological and biochemical processes.

### 2.2 Exposure pathways of plant enrichment

The pathways of plant uptake of metal-based NPs mainly include roots, leaves and other ways, which are affected by various factors such as the physicochemical properties of metal-based NPs, environmental conditions, and plant species and size.

#### 2.2.1 Absorption of metal-based NPs by leaves

**Figure 3:**
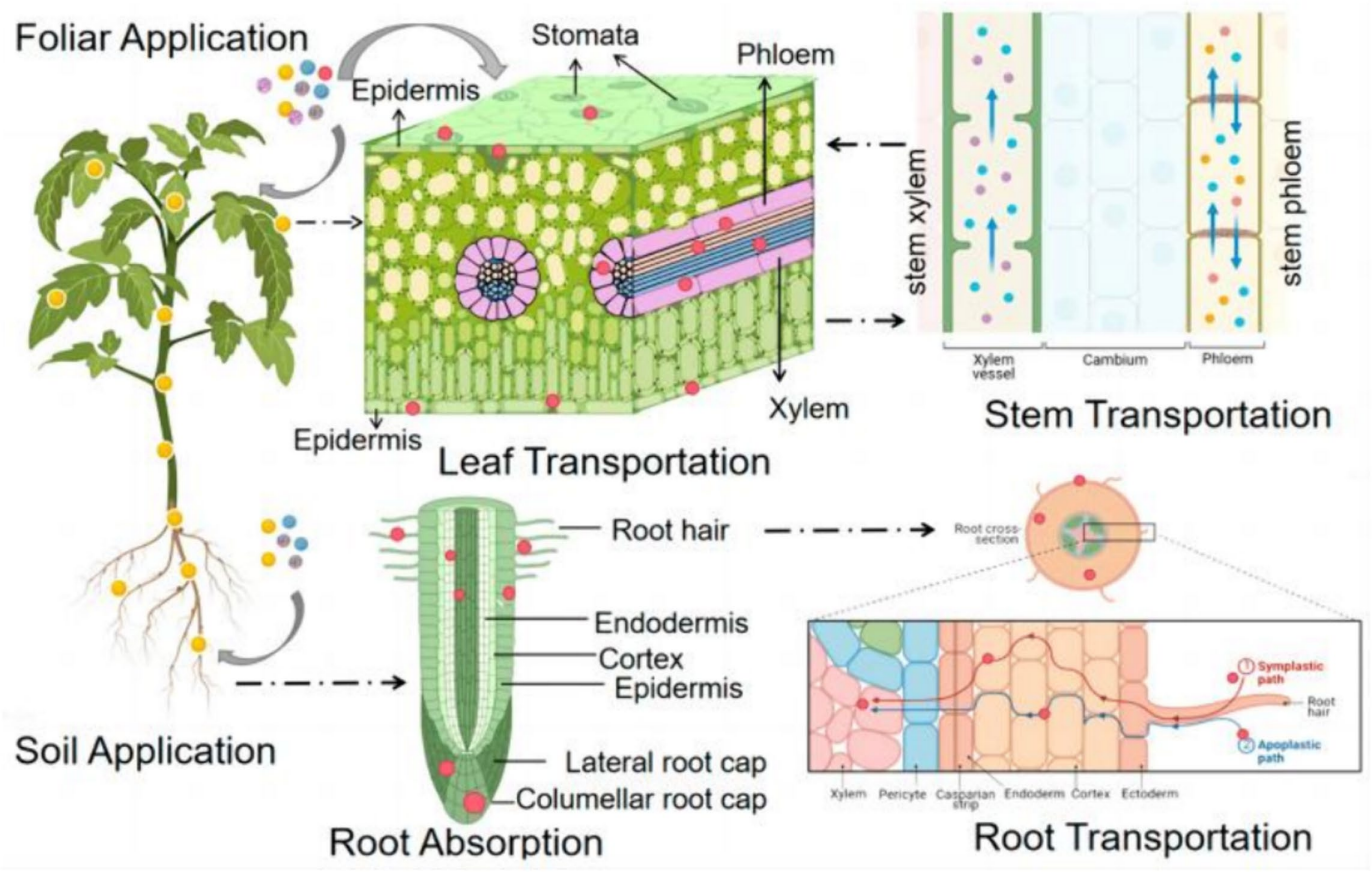
A schematic diagram of the uptake and translocation of NPs in plants through foliar application or root exposure treatment (24). Copyright 2023, Molecular Diversity Preservation International (MDPI).

Metal-based NPs can enter the plant through adsorption and penetration on the leaf surface. For example, studies on gold NPs (AuNPs) have shown that smaller-sized AuNPs (3, 10 nm) adhere more readily to leaf surfaces and are able to penetrate more efficiently through the epidermal layer into the plant compared to polyvinylpyrrolidone (PVP) coatings (23). In addition, the physicochemical properties of the NPs, such as size, surface charge, and chemical composition, affect their uptake and transport in the leaf (Figure 3) (24).

#### 2.2.2 Uptake of metal-based NPs by plant roots

Plant roots are another important pathway for metal-based NPs to enter the plant. The Fe(II) transporter protein encoded by the iron-regulated transporter (IRT1) gene was found in *Arabidopsis thaliana*, suggesting that plants can take up divalent Fe ions from roots via specific transporter proteins (24). In addition, some metal-based NPs, such as AgNPs, can also enter the plant via root uptake and may affect the physiological activity of the plant (25).

#### 2.2.3 Translocation of metal-based NPs in the plant vascular system

Once metal-based NPs enter the plant, they can be translocated through the plant’s vascular system. Studies have shown that metal-based NPs can be efficiently translocated from leaves to other parts of the plant, such as shoots and roots (23). This process may involve complex mechanisms within the plant, including metal transport involving organic molecules (26).

### 2.3 Exposure pathways of human and animal enrichment

Animals are exposed to metal-based NPs in a variety of ways, including inhalation, oral and dermal contact. These exposure modes reflect the behavior of NPs in the environment and their migration pathways within the organism, as well as their potential impact on the health of the organism. Therefore, these different exposure pathways need to be considered when assessing the effects of NPs on animal health.

#### 2.3.1 Inhalation exposure to metal-based NPs

Inhalation is a primary means of exposure to metal-based NPs, particularly in occupational settings or laboratories, where individuals may inhale them through respiration (27). Inhalation toxicity is mainly dependent on the physical and chemical properties of NPs, such as particle size, shape, surface chemistry, and biological activity (28, 29). The inhalation toxicity of metal-based NPs is closely related to their particle size, as demonstrated by inhalation toxicity studies. Generally, NPs with smaller particle sizes are more likely to penetrate the cell membrane and enter the cell interior, thus causing greater harm to the human body. Here, we summarize the inhalation exposure to some metal-based NPs (Table 1).

**Table 1:** Inhalation exposure to some metal-based NPs.

Materials	Dose (mg)	Model	Typical effects	Ref.
In_2_O_3_	0.05–0.6	Rats	Lung damage	(30)
ZnO	0–1	Monkeys	Pulmonary inflammatory	(31)
La_2_O_3_	0.5–10	Rats	Alveolar proteinosis	(32)
NiO	0.1, 0.2	Rats	Alveolar macrophages damage	(33)
WC	10	Rats	Pulmonary toxicity	(34)
MnO_2_	15, 30	Rats	Altered spontaneous cortical activity	(35)
Fe_2_O_3_	0.014–0.128	Mice	DNA strand breaks	(36)

For instance, Zhu et al. (37) compared the toxic effects of iron oxide NPs of different sizes on the lungs and found that nanosized Fe_2_O_3_ particles increased the microvascular permeability and cell lysis in the lung epithelium and significantly interfered with coagulation parameters compared with submicron Fe_2_O_3_ particles. Another study found that the deposition distribution of AuNPs in the lungs was age independent, that AuNPs was mainly deposited in the lung bases and cleared by mucus, and that in the long term, the clearance of AuNPs in the lungs and secondary organs was mainly mediated by macrophages (38).

The production of industrially manufactured TiO_2_ NPs is on the rise, posing a growing threat of inhalation exposure to professionals and consumers. Kreyling et al. (39) investigated the 28-day biokinetic pattern of the inhaled nanoparticulate material TiO_2_ NPs and found that NPs are redistributed within the alveoli over a long period through alveolar macrophage-mediated scavenging and reentry into alveolar epithelial cells. In addition, significant time-dependent differences were found in the accumulation and clearance process of TiO_2_ NPs *in vivo* compared with aerosol particles of the same size. In addition, Kim et al. (40) conducted research on inhaled nanomixes and found that the removal of Silver NPs (AgNPs) followed a two-phase model with rapid and slow dissolution rates, while the removal of AuNPs could be described by a single-phase model with a longer half-life. When exposed to both AuNPs and AgNPs, it was observed that the removal of AgNPs was affected by the presence of AuNPs. This change may be due to various interactions between AgNPs and AuNPs that influenced the solubilization and/or mechanical removal of AgNPs *in vivo*. After inhalation exposure, a minor proportion of the inhaled AgNPs dose that reaches the lungs is rapidly eliminated within the initial 72 h. The remaining portion of the dose is then slowly excreted. It appears that the inhaled dose cleared from the lungs is transferred to the body’s circulation between 48 and 72 h after inhalation (41).

#### 2.3.2 Oral ingestion exposure to metal-based NPs

**Figure 4:**
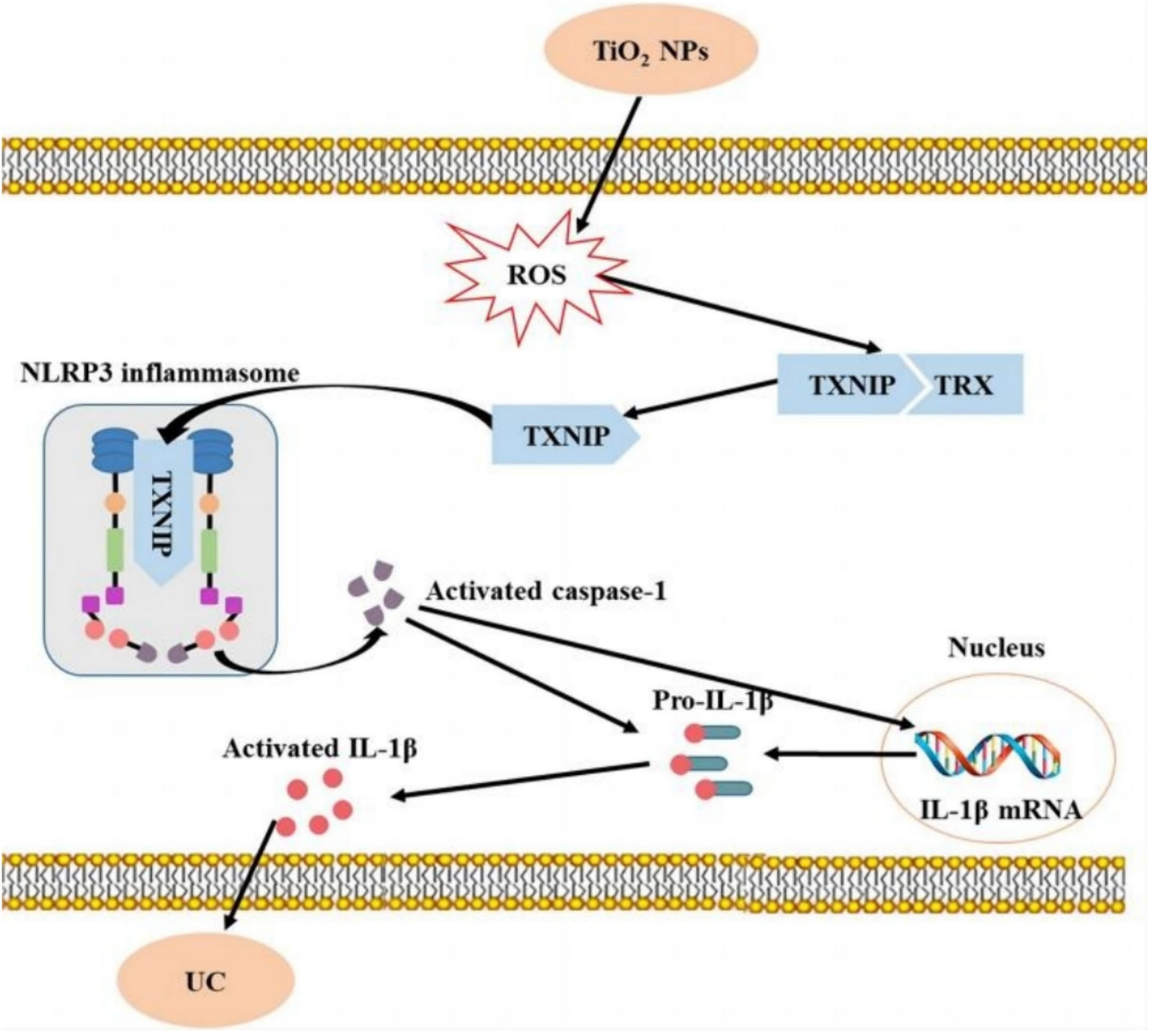
Short-term intake of TiO_2_ NPs induces mild colitis and exacerbates the development of ulcerative colitis (45). Copyright 2023, Springer Nature.

Metal-based NPs may be ingested during production and use, especially in food and pharmaceuticals. After oral ingestion of metal-based NPs, they may adhere to the gastrointestinal tract mucosa, causing local inflammation, ulcers, and other adverse reactions, and enter the blood system, causing damage to other organs and tissues (42–44). For example, some studies have shown that oral administration of TiO_2_ NPs, which are commonly used as food additives in candies, chocolates, and beverages, can affect the course of acute colitis and exacerbate the onset, prolong the course, and inhibit the recovery of ulcerative colitis (Figure 4) (45).

By contrast, Jones et al. (46) examined the gastrointestinal absorption of NPs in humans and *in vitro* using titanium dioxide as a model compound. They compared the behavior of NPs with larger particles and found no evidence that TiO_2_ NPs were more easily absorbed into the gut than micron-sized particles. Tang et al. (47) compared the detailed toxicity of copper NPs with CuCl_2_∙2H_2_O (copper ions) *in vivo*. They also examined the oral toxicity of four sizes of copper particles (30 n, 50 nm, 80 nm, and 1 μm) in rats. The researchers compared acute LD50 values of CuCl_2_∙2H_2_O and other copper materials under acute exposure. After administering a single equivalent dose (200 mg/kg) of five copper materials, researchers evaluated the kinetics of copper and found that the acute toxic effects produced by Cu NPs were strongly associated with particle size. Furthermore, repeated exposure to copper NPs produced toxic effects that differed from those observed with single exposure. The size of the NPs may be responsible for the organ-targeting effects. This could explain the observed differences in organ-specific accumulation. Here, we summarize the Oral ingestion exposure to some metal-based NPs (Table 2).

**Table 2:** Oral ingestion exposure to some metal-based NPs.

Materials	Dose (mg)	Model	Typical effects	Ref.
TiO_2_	0–300	Mice	Prolonging the UC course	(45)
Cu	60–180	Rats	Fetal development	(48)
MgO	250–1,000	Rats	Genotoxicity	(49)
Y_2_O_3_	30–480	Rats	Apparent genotoxicity	(50)
NiO	500–1,000	Rats	Metabolic abnormality	(51)

### 2.4 Dermal exposure to metal-based NPs

Metal-based NPs may have irritating effects on the skin and cause skin inflammation and allergic reactions. Some studies have shown that these NPs may adhere to the skin surface, have toxic effects on skin cells, and induce skin inflammation and allergic reactions. In addition, metal-based NPs may enter the body through broken skin and cause damage and irritation to deeper skin cells and tissues (52, 53). AuNPs are used for many applications, but available data are lacking on their dermal absorption. Filon et al. (54) conducted experiments utilizing the Franz diffusion cell technique to examine the penetration of intact and compromised human skin by AuNPs. Their findings revealed that AuNPs are capable of permeating human skin in an *in vitro* diffusion cell system. The growing utilization of palladium NPs (PdNPs) in various chemical processes, jewelry production, electronic gadgets, automotive catalytic converters, and medical uses has resulted in a notable rise in palladium exposure. Exposure of the skin to palladium can lead to allergic contact dermatitis. For example, Filon et al. (55) found that PdNPs can significantly penetrate the skin.

## 3 Toxic effects of metal-based NPs

The widespread use of metal-based NPs has also led to their potential toxic effects on organisms. Such ecotoxicity effects are closely related to factors such as the type, size, surface properties, and concentration and exposure duration of NPs. Herein, we summarize various ecotoxicity effects such as toxicity to aquatic organisms, plants, animals and human.

### 3.1 Toxicity of metal-based NPs to aquatic organisms

In recent years, scholars have begun to focus on the toxic effects of metal-based NPs on aquatic organisms, and have achieved certain results. Current studies have mainly concentrated on the toxic effects of metal-based NPs on aquatic animals. However, research has shown that these NPs have various effects on aquatic organisms (56–58). The toxic effects of metal-based NPs on aquatic organisms are complex and diverse. The degree of toxicity varies depending on the type of metal-based NPs, with each type possessing unique physical, chemical, morphological, and biological characteristics that influence their impact on aquatic organisms.

#### 3.1.1 Toxicity to fish

**Figure 5:**
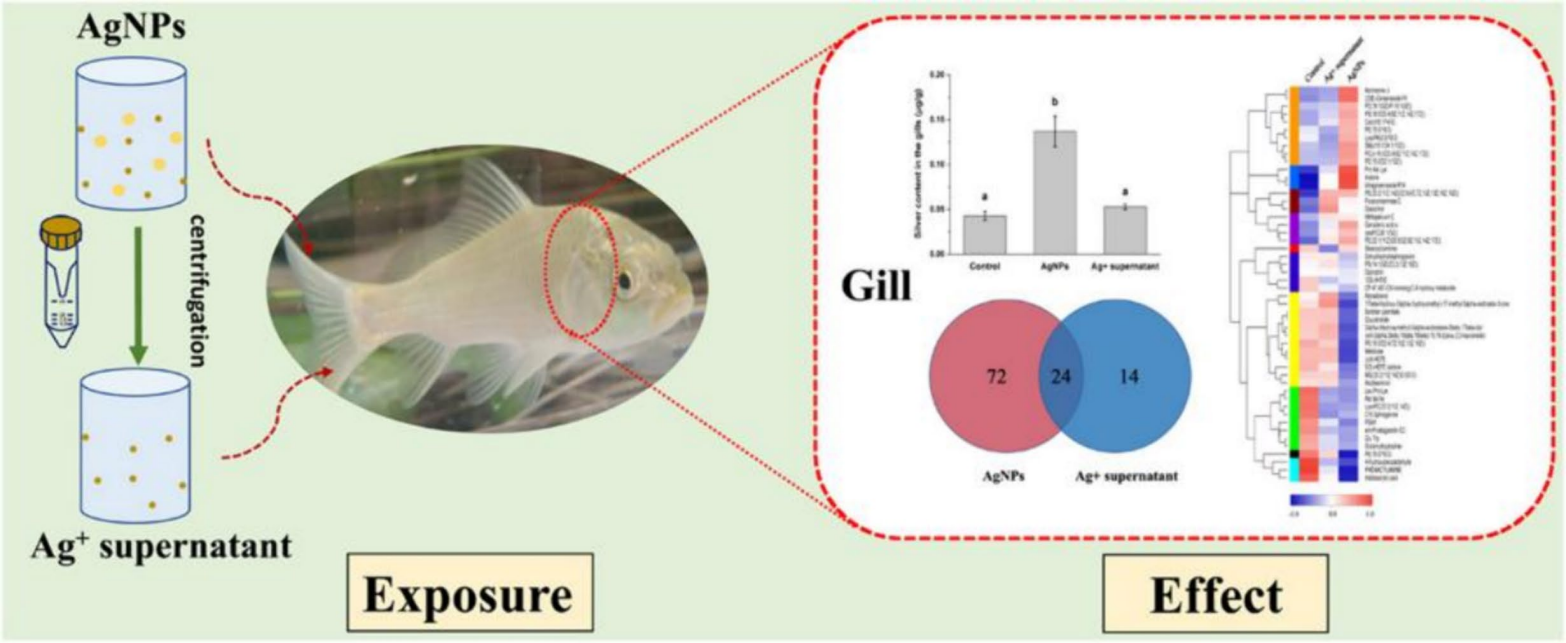
Schematic diagram of nano-silver toxicity in carp gills (60). Copyright 2021, Elsevier.

Studies have shown that the amount of NPs in the water column and the form in which they are present in the water column can have an effect on fish. Marinho et al. (59) conducted an analysis on the impact of exposure to various AgNPs concentrations on zebrafish tissues, discovering a substantial reduction in acetylcholinesterase (AChE) activities in both the brain and muscle. Another study observed that exposure to AgNPs decreased levels of l-histidine, l-isoleucine, and l-phenylalanine, crucial amino acids in fish gills. This suggests that AgNPs may disrupt amino acid metabolism, potentially affecting fish health and function. Furthermore, AgNPs altered citric acid levels, possibly disrupting the citrate cycle, essential for energy production. This disruption could lead to decreased energy production and metabolic dysfunction in fish gills. The present findings stress the potential consequences of AgNPs on fish metabolism, emphasizing the requirement for more research on the effects of NP exposure on aquatic lifeforms (Figure 5) (60). Another study on TiO_2_ NPs revealed that the treatment dose of these NPs was directly linked to increased motility and bacterial population in water. Notably, the zebrafish exhibited a significant rise in the bacterial load in its gills and caudal fins (61).

#### 3.1.2 Toxicity to shellfish

As an important component of aquatic animals, the health status of shellfish is of great significance in maintaining the stability of the entire ecosystem. Shellfish have a strong bioconcentration effect on heavy metals and other pollutants and show different degrees of enrichment patterns in different sea areas. Elevated levels of ZnO NPs had a significant impact on various physiological parameters in the thick-shelled mussel, *Mytilus coruscus*. These effects included a decrease in total hematocrit, phagocytosis, esterase, and lysosomal contents, as well as an increase in hematocrit and ROS levels. Furthermore, the combination of high ZnO NPs concentrations and low pH had a negative synergistic effect on the mussels (62). AgNPs are frequently used in consumer products due to their antimicrobial and exceptional properties, leading to increasing concerns about their potential impact on aquatic ecosystems. Duroudier et al. (62) found that PVP/PEI-coated AgNPs ingested through the food web accumulated significantly in mussel tissues and adversely affected cell and tissue levels in autumn and spring. Furthermore, the total hematocrit, phagocytosis, esterase, and lysosomal contents of mussels were found to decrease at low pH and elevated concentrations of TiO_2_ NPs. Conversely, the hematocrit and ROS levels were observed to increase with increasing TiO_2_ NPs concentration under low pH conditions (63). The majority of recent studies have primarily concentrated on the toxic effects of individual metal NPs on mussels. However, further research is required to comprehensively examine the toxic impact of metal NPs on mussels as a whole.

### 3.2 Toxicity of metal-based NPs to plants

In recent years, the ecotoxicological response of plants to NPs has gradually become a research topic. The toxicity of metal-based NPs to plants is mainly manifested in two aspects: plant growth inhibition and the influence of plant metabolic processes.

#### 3.2.1 Plant growth inhibition

**Figure 6:**
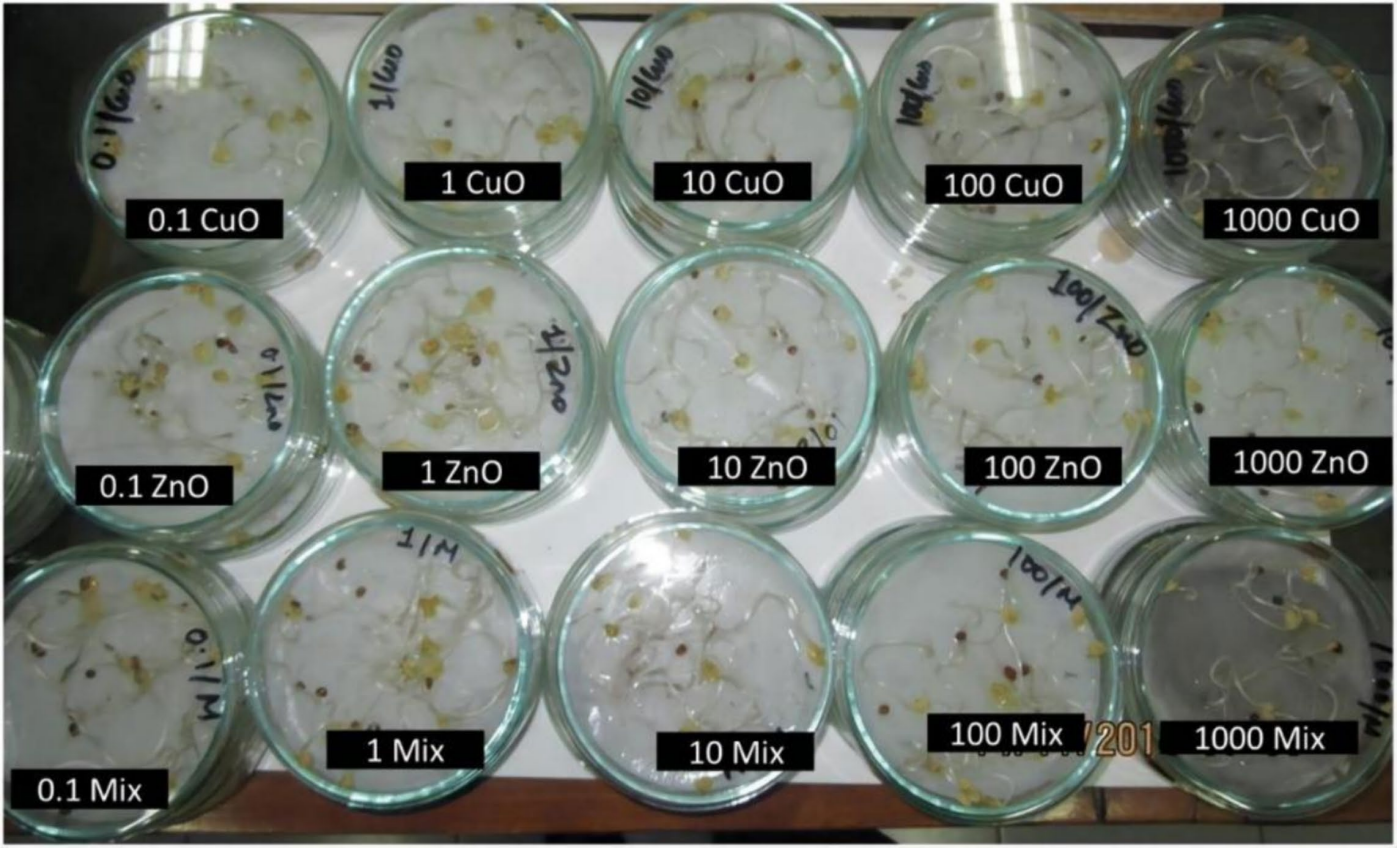
Images showing radish seedlings exposed to varying concentrations of different NPs (68). Copyright 2019, Springer Nature.

Plant growth is affected by several factors, including soil, temperature, moisture, and light. Although soil is the most significant factor impacting plant growth, certain NPs can also exhibit inhibitory effects on plants. During the early growth stage, the inhibitory effect of NPs on plants is primarily manifest as a suppression of germination and seedling development (64, 65). For example, Zhang et al. (66) carried out research into the influence of ZnO NPs on the germination of seeds and the growth of roots in maize and cucumber. Their findings indicated that the inhibitory effect of ZnO NPs on root growth in maize was predominantly attributed to the NPs, as opposed to the Zn^2+^ ions. Conversely, the Zn ions released from ZnO only inhibited root elongation in cucumber. The toxicity level of ZnO NPs was found to be dependent on its concentration (67). The phytotoxicity ranking shows that CuO NPs have the highest toxicity, followed by the binary mixture (CuO + ZnO) NPs, and then ZnO NPs. This significant toxicity and uptake in germinating seedlings is observed when exposure concentrations exceed 10 mg/L (Figure 6) (68).

#### 3.2.2 Influence on plant metabolic processes

**Figure 7:**
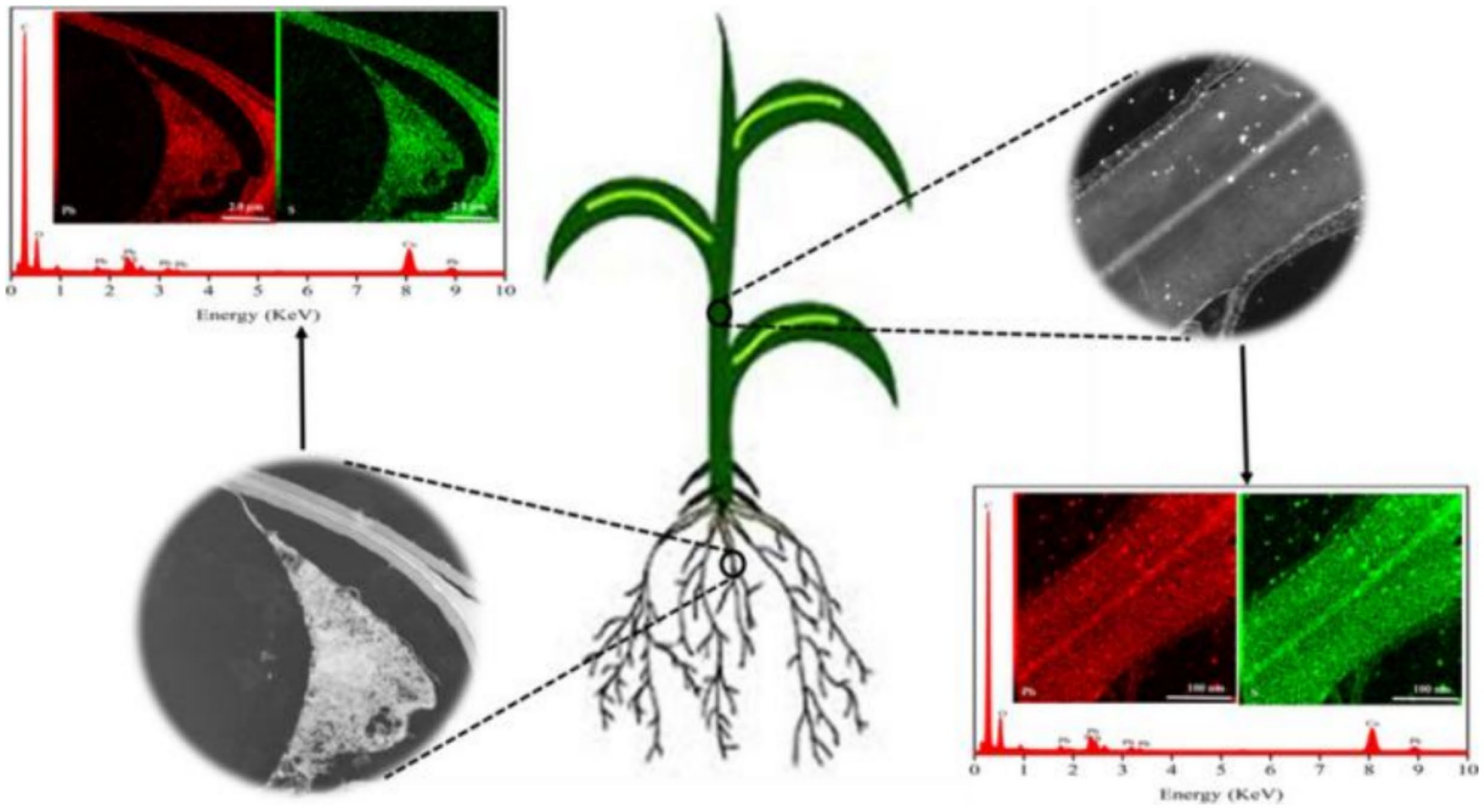
Diagram of the uptake of PbS NPs in plants (70). Copyright 2020, Elsevier.

When metal-based NPs are introduced into plants, they enter the cell and affect plant metabolic processes by altering the intracellular environment. Chloroplasts, mitochondria, and peroxisomes, which have high oxidative metabolic activity and electron flow rates, are the primary sources of ROS in plant cells. The production of ROS by these organelles can lead to lipid peroxidation, membrane fluidity and permeability changes, and nutrient acquisition difficulties, ultimately impeding overall plant growth and development. NPs can also affect these processes, causing further damage to plant cells (69). In addition, metal-based NPs can affect the metabolites of secondary metabolites such as amino acids (Figure 7) (70). NPs have the potential to induce DNA damage, including DNA mismatch damage, DNA strand breaks, and chromosome damage. TiO_2_ NPs are known to be especially detrimental in this regard (70).

### 3.3 Toxicity of metal-based NPs to animals

The toxicity of NPs can be attributed to their physicochemical properties, such as size, surface chemistry, and redox potential, and is associated with the dissolution and release of toxic metals. Metal-based NPs are significantly toxic to human, including to the immune system (48, 71, 72).

For example, metal-based NPs can cause structural and functional damages to the ovary and testis. One research study discovered that Cu NPs induced both intrinsic and extrinsic apoptotic pathways in oxidative stress-induced ovarian dysfunction and controlled important ovarian genes, leading to harm to ovarian tissue (73). Subsequent study has shown that Cu NPs are a greater threat to reproduction than copper particles. This is due to the direct damage caused by Cu NPs to the ovary and their impact on ovarian hormone metabolism (74). Yang et al. (75) discovered that exposure to CdSe/ZnS quantum dots impairs the repair of double-strand breaks in spermatocytes, disrupts meiotic progression, and causes apoptosis and reduced sperm production.

Indeed, the potential for NPs to cross the alveolar-capillary barrier and enter the bloodstream, thereby reaching other organs, is a legitimate concern. For example, Nemmar et al. (76) discovered that mice exposed to CeO_2_ NPs exhibited a dose-dependent infiltration of inflammatory cells, including macrophages and neutrophils, in their lung sections. These findings suggest that acute lung exposure to CeO_2_ NPs triggers pulmonary and systemic inflammation, oxidative stress, and promotes *in vivo* thrombus formation. Similarly, TiO_2_ NPs exhibit size-dependent genotoxicity, with smaller particles being more significantly toxic (77). Kim et al. (30) found that a single inhalation exposure to anosized indium oxide (In_3_O_2_) resulted in worsening of lung damage such as chronic active inflammation, foamy macrophage infiltration, and granulomas. Early-onset and persistent pulmonary alveolar proteosis, even at very low doses, indicates an urgent need to reassess occupationally recommended exposure limits for In_3_O_2_ NPs to protect workers.

Compared with ordinary metal ions, metal-based NPs are more likely to penetrate into cell membranes or cells, causing excessive generation of intracellular superoxide anions, damaging membrane integrity and thus causing oxidative damage leading to cell death, and resulting in toxic effects on the digestive and nervous systems, among others (78, 79).

### 3.4 Toxicity of metal-based NPs to human

These metal-based NPs, particularly noble metals such as gold, silver and platinum, have shown significant potential in the treatment of various diseases, including cancer, pneumonia and Parkinson’s disease, due to their unique optoelectronic properties and ease of surface functionalisation (80, 81).

However, metal-based NPs can enter the human body through multiple pathways and affect different tissues and systems. Its toxic effects are multifaceted and include effects on the immune system, cytotoxicity and genotoxicity. For example, copper oxide NPs are able to activate the production of reactive oxygen species and pro-inflammatory cytokines in human lung epithelial cells (82), whereas silver, gold, and platinum NPs can enter the human body through therapeutic applications and cause damage to erythrocytes, including hemolysis, agglutination, and membrane damage (83). In addition, metal-based NPs can affect the systemic system by being deposited through the respiratory tract and taken up by phagocytes in the lung (84). It can enter the human body through skin exposure, and although the skin barrier prevent the penetration of NPs to some extent, it has been shown that NPs are able to cross the skin barrier under certain conditions (85, 86).

Notably, the morphology of metal-based nanoparticles has a significant effect on the toxicity of skin pathogens and HaCaT keratinocytes. It was shown that the toxicity of different shapes of AgNPs to bacteria and HaCaT cells varied, with truncated plate-shaped AgNPs showing the highest cytotoxicity (87). The biodistribution and metabolic consequences of metal-based NPs have also been the focus of research. Several studies have shown that metal-based NPs can migrate *in vivo* to locations far from the site of administration, requiring careful monitoring of their migration pathways and potential toxic effects (88). For example, inhaled ultrafine manganese oxide NPs can migrate to the central nervous system via the olfactory nerve pathway, causing inflammatory changes (89).

**Figure 8:**
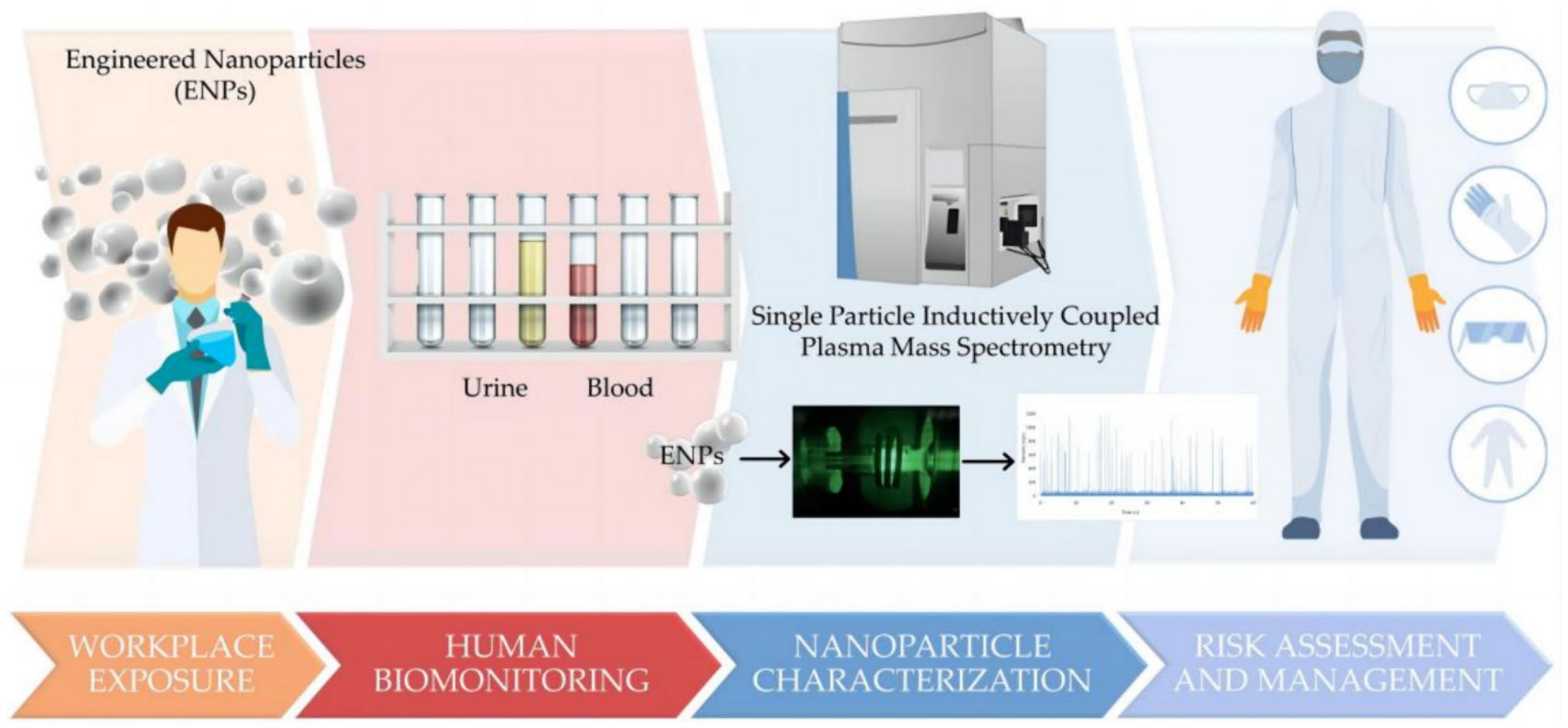
A human biomonitoring method based on single particle inductively coupled plasma mass spectrometry (90). Copyright 2023, Molecular Diversity Preservation International (MDPI).

For human exposure assessment of metal-based NPs, a comprehensive approach is needed to consider their safety. For example, a study of Italian nanomaterials workers developed a human biomonitoring method based on single-particle inductively coupled plasma mass spectrometry to assess the level of NPs exposure in the workplace (Figure 8) (90).

## 4 Toxicity mechanisms of metal-based NPs

The mechanism of toxicity for metal-based NPs is multifaceted and intricate. In terms of the interaction between NPs and living organisms, the size and shape of metal-based NPs have a significant impact on their interactions with cells. For instance, smaller NPs tend to accumulate more easily in cells, potentially causing damage to cellular structures and disrupting normal cell function. Furthermore, the surface properties of metal-based NPs can influence their interactions with proteins and other biomolecules, leading to adverse effects on cell health. Therefore, a better understanding of the mechanisms underlying the toxicity of metal-based NPs is essential for the development of effective safety measures and the design of more biocompatible materials (91, 92).

### 4.1 Cell membrane damage

The cell membrane is a barrier for the exchange of substances inside and outside the cell, preventing harmful substances from entering the cell and protecting the internal structure of the cell. Studies have shown that metal-based NPs may cause direct damage to the cell membrane, resulting in altered cell membrane permeability (93), the disruption of cell membrane integrity (94), and the alteration of cell membrane structure (95), among others. For example, zinc oxide NPs induce toxicity by affecting cell wall integrity pathways, mitochondrial function, and lipid homeostasis in *Saccharomyces cerevisiae* (96). Chen et al. (12) studied the biological effects of TiO_2_ NPs on the unicellular green alga *Chlamydomonas reinhardtii*. The cell surface morphology of *Chlamydomonas reinhardtii* was found to be altered on scanning electron microscopy, indicating that photocatalytic TiO_2_ NPs disrupted the cell surface.

### 4.2 Intracellular oxidative stress

In a normal environment, intracellular ROS are generated at a low production rate and rapidly eliminated by antioxidant defense systems such as glutathione and antioxidant enzymes, thus maintaining cellular redox balance. However, when ROS are overproduced, the redox reaction becomes unbalanced, triggering a series of biochemical reactions that lead to cellular damage (97, 98). The mechanism of action of metal-based NPs is, on the one hand, to increase the production of ROS, and the generation of excess ROS is the precursor to oxidative damage effects. Direct contact of NPs with the mitochondria or storage in the acidic environment of lysosomes allows for the direct cellular production of ROS (99, 100). On the other hand, metal-based NPs cause the intracellular antioxidant enzyme system to be underproduced. The antioxidant enzyme system includes superoxide dismutase, catalase, and glutathione peroxidase (101, 102). For example, when Ag NPs are used as a stressor, *Cryptobacterium hidradii* nematodes can regulate oxidative stress through the p38 MAPK pathway (103).

### 4.3 Cellular inflammation

NF-κB-regulated inflammatory response plays an important role in the differentiation, value addition, and expression of biological proteins and biological enzymes. When mouse hearts were exposed to TiO_2_ NPs, cardiomyocyte swelling and inflammatory cell infiltration were observed, as a significant increase in NF-κB promoted the expression of IL-1β and TNF-α (104). Another study revealed that ZnO NPs play an important role in regulating the inflammatory response of vascular endothelial cells through NF-κB signaling, which may be important for the treatment of vascular diseases (105). The inflammatory response of ZnO NPs was also confirmed in another study (106). In addition, metal oxide NPs can activate human lung epithelial cells to produce ROS and pro-inflammatory cytokines such as interleukin 8 and granulocyte-macrophage colony-stimulating factor, which activate and recruit immune cells (82).

### 4.4 Regulation of gene expression

Abnormalities in gene expression levels can be caused by mutations, environmental factors, or dysregulation of intracellular regulatory mechanisms (107, 108). For example, metal-based NPs may interfere with gene transcription, affecting the binding of DNA to RNA polymerase, leading to abnormal gene transcription, which in turn affects protein expression and function (109). Alternatively, they may affect the DNA methylation status, which in turn affects the regulation of gene expression. Methylation is an important mode of gene expression regulation, and metal-based NPs may affect gene expression and function by altering the DNA methylation state (13).

## 5 Challenges and prospects for the ecotoxicity of metal-based NPs

Some progress has been made in the research on the ecotoxicity of metal-based NPs, but there are still many challenges and problems to be solved. First, the ecotoxicity assessment of metal-based NPs requires an integrated assessment approach. Integrated biomarker response has been shown to be an effective tool for assessing the toxic effects of metal-based NPs on environmental biomass. In addition, computational toxicology applications such as quantitative structure–activity relationships and read across techniques are important for predicting nanotoxicity and filling data gaps. Second, it is necessary to strengthen the research on the interactions and mechanisms between metal-based NPs and living organisms, including their direct effects on living organisms and potential risks. In addition, experimental studies and field investigations should be actively conducted to assess the potential impacts of metal-based NPs on the environment and human health.

In order to manage the ecotoxicity risks of metal-based NPs, appropriate regulatory measures need to be developed. This includes the classification and labelling of nanomaterials and the setting of hazard threshold levels for human health and the environment. Furthermore, research should focus on increasing the body’s resistance to the harmful effects of metal-based nanoparticles in order to mitigate their potential toxic effects.

To achieve this goal, interdisciplinary collaboration is essential, involving researchers from a wide range of fields, including chemistry, physics, biology, and environmental sciences, to promote the in-depth development of ecotoxicity research on metal-based NPs. Looking ahead, with continuous progress and innovation in science and technology, we are confident that the impacts of metal-based NPs on the environment and human health can be better understood and controlled. At the same time, there is a need to strengthen public education on scientific literacy, improve public awareness and understanding of nanotechnology, and promote the sustainable development and application of nanotechnology.

## Author contributions

FW: Formal analysis, Investigation, Writing – review & editing, Writing – original draft. LZ: Data curation, Resources, Writing – review & editing, Writing – original draft. DM: Data curation, Writing – review & editing. HZ: Formal analysis, Writing – review & editing. GZ: Conceptualization, Resources, Writing – review & editing. XH: Conceptualization, Resources, Writing – review & editing. PX: Conceptualization, Resources, Writing – review & editing, Writing – original draft.
